# Perspective of SGLT2 Inhibition in Treatment of Conditions Connected to Neuronal Loss: Focus on Alzheimer’s Disease and Ischemia-Related Brain Injury

**DOI:** 10.3390/ph13110379

**Published:** 2020-11-11

**Authors:** Michał Wiciński, Eryk Wódkiewicz, Karol Górski, Maciej Walczak, Bartosz Malinowski

**Affiliations:** Department of Pharmacology and Therapeutics, Faculty of Medicine, Collegium Medicum in Bydgoszcz, Nicolaus Copernicus University, M. Curie 9, 85-090 Bydgoszcz, Poland; wicinski4@wp.pl (M.W.); karolgorski-2@gazeta.pl (K.G.); maciej.walczak5@hotmail.com (M.W.); bartosz.malin@gmail.com (B.M.)

**Keywords:** pharmacology, neurodegeneration, cytokines, Alzheimer’s disease, stroke

## Abstract

Sodium-glucose co-transporter 2 inhibitors (SGLT2i) are oral anti-hyperglycemic agents approved for the treatment of type 2 diabetes mellitus. Some reports suggest their presence in the central nervous system and possible neuroprotective properties. SGLT2 inhibition by empagliflozin has shown to reduce amyloid burden in cortical regions of APP/PS1xd/db mice. The same effect was noticed regarding tau pathology and brain atrophy volume. Empagliflozin presented beneficial effect on cognitive function, which may be connected to an increase in cerebral brain-derived neurotrophic factor. Canagliflozin and dapagliflozin may possess acetylcholinesterase inhibiting activity, resembling in this matter Alzheimer’s disease-registered therapies. SGLT2 inhibitors may prove to impact risk factors of atherosclerosis and pathways participating both in acute and late stage of stroke. Their mechanism of action can be related to induction in hepatocyte nuclear factor-1α, vascular endothelial growth factor-A, and proinflammatory factors limitation. Empagliflozin may have a positive effect on preservation of neurovascular unit in diabetic mice, preventing its aberrant remodeling. Canagliflozin seems to present some cytostatic properties by limiting both human and mice endothelial cells proliferation. The paper presents potential mechanisms of SGLT-2 inhibitors in conditions connected with neuronal damage, with special emphasis on Alzheimer’s disease and cerebral ischemia.

## 1. SGLT2 Receptor May Be a Potential Target in Therapies of CNS Disorders

**Figure 1:**
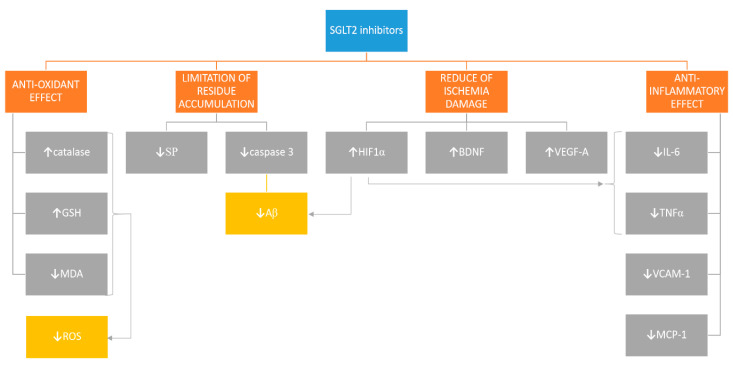
Proposed mechanisms of Sodium-glucose co-transporter 2 inhibitors (SGLT2i) activity. ↓—reduction, ↑—increase, GSH—glutathione, MDA—malondialdehyde, ROS—reactive oxygen species, SP—senile plaques, Aβ—amyloid β, HIF1α—hypoxia-inducible factor 1α, BDNF—brain-derived neurotrophic factor, VEGF-A—vascular endothelial growth factor-A, IL-6—interleukin 6, TNFα—tumor necrosis factor α, VCAM-1—vascular cell adhesion protein, MCP-1—monocyte chemotactic protein-1. The differences in colors of Figure 1 have been used for aesthetic purposes only.

Sodium-glucose co-transporter 2 inhibitors (SGLT2i) are oral anti-hyperglycemic agents approved for the treatment of type 2 diabetes mellitus [1]. Sodium-glucose co-transporter 2 (SGLT2) is expressed primarily in segments 1 and 2 of the proximal convoluted tubule in the kidney. It plays an important role in the reabsorption of urinary glucose, which is dependent on a sodium concentration gradient [2]. SGLT family of co-transporters contains membrane spanning monomer proteins with 14 transmembrane domains and a single N-glycosylation site. SGLTs transport glucose and galactose against a concentration gradient with simultaneous transport of Na^+^ ions [3]. The main mechanism of their action is based on increase of glucose excretion through a reduction in renal reabsorption. The diuretic glycosuric, and natriuretic properties of these compounds increase volume and sodium load of urine [2]. There is an increasing number of reports stating sodium-glucose co-transporters (SGLTs) presence in mammalian central nervous system (CNS) [4,5]. It has been proved that the SGLT1 receptor is expressed in such areas as CA1, CA3 (region 1 and 3 of hippocampal cornu ammonis), and the dentate gyrus hippocampal subfields, while significant expression of SGLT2 has been identified in the hippocampus, cerebellum, and at blood–brain barrier (BBB) endothelial cells [3,6,7,8]. The search for effective neuroprotective drugs to help treat acute and chronic brain diseases has been going on for years. It seems that this particular distribution may be responsible for the intriguing evidence suggesting their neuroprotective properties [9]. Proposed mechanisms of SGLT2i activity are presented in Figure 1.

In the EU, three SGLT2i are currently authorized and will be discussed in the paper: canagliflozin (Invokana and Vokanamet), dapagliflozin (Forxiga and Xigduo), and empagliflozin (Jardiance and Synjardy). All three compounds have shown to be more effective than placebo at reducing HbA1c (glycated hemoglobin) levels. Apart from glucose lowering, SGLT2 inhibitors presented other beneficial and clinically relevant metabolic effects, such as improvements in fasting plasma glucose (FPG), body weight, and systolic and diastolic blood pressure [10,11]. Based on current evidence, SGLT2 inhibitors reduce the risk of cardiovascular events, especially in patients with heart failure [12]. Empagliflozin has proved to possess higher selectivity for SGLT2 (2500-fold) than dapagliflozin (1200-fold) and canagliflozin (250-fold) [13]. Genital infections appear to be the most common adverse side effect (4-fold increase) [14]. SGLT2 inhibition induces osmotic diuresis, which might lead to volume depletion. Risk factors for the adverse effect are: age > 75 years, GFR (glomerular filtration rate) < 60 mL/min/1.73 m^2^, and use of loop diuretics [15]. Analysis performed by European Medical Agency presented 102 cases of serious diabetic ketoacidosis in patients on SGLT2 inhibitor treatment (canagliflozin, dapagliflozin, or empagliflozin) for T2DM (diabetes mellitus type 2). Interestingly, some of them occurred to be atypical, with moderately increased blood glucose levels [16,17,18,19,20]. Unfortunately, current information on the matter relies on poorly documented cases, which severely limits the possibility for appropriate assessment and identification of risk groups.

## 2. SGLT2i Are Another DM-Registered Treatment That May Have an Influence on Alzheimer’s Disease Pathology

Alzheimer’s disease (AD) is the sixth-leading reason for fatality, consisting of over two-thirds of all cases of dementia. The global burden of AD is expected to grow from 26.6 million cases in 2006 to 106.8 million by 2050 [21,22]. Extent of these data and undoubted disappointment with the effects of therapeutic solutions imply a substantial need for new efficient options in treatment of broadly defined dementia.

One of the major pathological mechanism of AD is accumulation of extra- and intracellular residues called respectively plaques-built of beta-amyloid and neurofibrillary tangles (NFTs)—aggregates of tau protein that bind to microtubules [23,24]. Recent studies suggest that SGLT inhibition may have some impact on the process. Hierro-Bujalance et al. [25], in the first of its kind study regarding SGLT2 inhibitor—empagliflozin (EMP) reportedly reduced senile plaque density and overall reduction of soluble and insoluble amyloid β (Aβ) levels in the cortex and hippocampus of EMP-treated mice of APP/PS1xd/db model. In APP/PS1xd/db mice, brain atrophy is observed as the disease progresses, making the model resembling actual AD pathology. The similar effect in amelioration of amyloid pathology has been observed due to other diabetes mellitus (DM)-registered treatment [26,27,28], based not only on metabolic control, but also on their effects on oxidative stress, inflammation, or the blood–brain barrier (BBB). The second kind of AD characteristic aggregates has also appeared to be limited due to EMP administration. Although it helped to reduce tau phosphorylation in the cortex, statistical significance in the matter has not been reached in the hippocampus area opposite to incretin-based DM treatment results [29,30]. The same discrepancy has been observed in brain atrophy extent where statistical significance has been observed only in the cortical region. What is more, authors reported beneficial effect regarding cognitive function in new object discrimination task and Morris water maze testing where EMP treatment positively affected cognitive abilities of APP/PS1xdb/db mice and significantly ameliorated memory impairment. The researchers could not exclude the leptin signaling alteration as a cause of improvement [25]. In the work of Lin et al. [10], empagliflozin significantly prevented cognitive function impairment in db/db mice, which was associated with the attenuation of cerebral oxidative stress and the increase in cerebral brain-derived neurotrophic factor (BDNF). BDNF supports differentiation, maturation, and survival of neurons in the nervous system [23], and presents neuroprotective properties under such conditions as: glutamatergic stimulation, cerebral ischemia, hypoglycemia, and neurotoxicity [31,32]. This mechanism has been previously proposed as responsible of neuroprotective properties of glucagon like peptide 1 agonist-liraglutide [33,34].

Although the efficacy of FDA-registered AD-dedicated treatments leaves much to be desired, acetylcholinesterase inhibitors and N-methyl-D-aspartate receptor antagonists remain the leading therapeutical options for the suffering patients. In scopolamine-induced memory impairment rat model of Arafa et al. [35] researchers observed beneficial effect of another SGLT2 inhibitor called canagliflozin. They attribute this effect to possible acetylcholinesterase inhibiting activity of the drug as an additional property of SGLT inhibitors. The findings seem to be consistent with the results of molecular docking technique studies evaluating the binding energy of dapagliflozin, and acetylcholinesterase’s (AChE) 19 amino acid residues, which determine the strength of interaction between an inhibitor and an enzyme [36]. The lowest binding energy is the mark of the best conformer at its receptor site or active site of an enzyme [37]. The docked ligand presented acceptable binding energy values, i.e., −6.28 kcal/mol and −6.25 kcal/mol for AChE and SGLT2 [36], implying possible dapagliflozin dual inhibitor properties and reaffirming the results of Rizvi et al. and Shakil, also in other SGLTi, such as ertugliflozin and sotagliflozin [38,39].

## 3. SGLT2i May Prove to Enhance Angiogenesis and Neurogenesis and Prevent Ischemia-Related Cerebral Damage

Cerebral ischemic stroke is one of the leading causes of death and long-term disability in the world [40]. Blood flow obstruction leads to the development of pathophysiological changes and ultimately to tissue necrosis and neuronal loss [41]. Abdel-Latif et al. [42] found that empagliflozin, at both low and high doses, attenuated neurological defects in rats of induced ischemia model utilizing occlusion of the carotid arteries. It is noteworthy that beneficial effect of empagliflozin seemed to be dose-dependent, presenting higher neurological scores in groups administered with higher dosage. Empagliflozin increased the level of hypoxia-inducible factor 1α (HIF-1α) and the expression of the vascular endothelial growth factor A (VEGF-A) protein, decreased the expression of caspase-3 and limited infarct volume compared to the control group. In order to prevent or reduce the negative effects of ischemia, signaling pathways and factors are activated in hypoxia-affected cells. A representative of such agents is HIF-1α. It supports the restoration of oxygen homeostasis by inducing glycolysis, erythropoiesis and angiogenesis [43]. The results obtained by Xing et al. [44] indicate that HIF-1α activation significantly weakened the increased expression of the interleukin-6/interleukin-6 receptor (IL-6/IL-6R) pathway, tumor necrosis factor α/tumor necrosis factor receptor 1 (TNF-α/TNFR1), and decreased the upregulated expression of caspase-3 in rat-induced global ischemia. The authors suggest that HIF-1α via reduction of proinflammatory cytokine expression can contribute to the limitation of detrimental brain ischemia effects [44]. In reference to the previous paragraph, caspase-3 overexpression may play several roles in AD pathogenesis involving amyloidosis [45], NFT formation [46], and neuronal apoptosis [47].

While the mechanisms underlying changes in SGLT2 expression in DM2 (diabetes mellitus type 2) are not well understood, changes in hepatocyte nuclear factor 1α (HNF-1α) and hepatocyte nuclear factor 3β (HNF-3β) activity have been reported to contribute to alterations in SGLT2 expression [48]. HNF-1α appears to directly control SGLT2 expression in mice and humans [49]. Researchers demonstrated that elevated HNF-1α expression and binding activity in the solute carrier family 5-member 2 promoter leads to the diabetes-induced overexpression of SGLT2 [50]. It is reasonable to hypostatize that above-mentioned example of EMP-induced elevation in HIF-1α may constitute a premise of certain feedback loop including SGLT-2 receptor and HNF-1α expression.

Another factor that may be responsible for protecting the cell from the effects of hypoxia and has been increased during EMP treatment is VEGF-A. VEGFs are known to be important regulators of angiogenesis and neurogenesis [51]. As a result of cerebral ischemic event VEGF-A and its receptors vascular endothelial growth factor receptor 1 and vascular endothelial growth factor receptor-2, are being upregulated [52]. In the brain of *Heterocephalus glaber* rodent species, expression of VEGF-A is significantly upregulated, which seems to contribute to their exceptional intrinsic tolerance to hypoxia [53]. On the other hand, activation of VEGF-A in the acute phase of stroke causes the breakdown of BBB, which leads to impaired homeostasis and, consequently, results in edema [54]. The moment of growth of VEGF-A level appears to be crucial during assessment of VEGF-A treatment efficacy. Unfortunately, the current state of knowledge concerning the role of VEGF-A in stroke is based mainly on research in animal models. 

Brain microvasculature has a close structural and functional relationship with brain parenchyma which is under control of biological system called neurovascular unit (NVU) [55]. NVU constitutes an integrative biological system of neurons, glial cells, and vascular cells in combination with extracellular matrix. Hayden et al. reported cognitive impairment, brain tissue oxidative stress, and ultrastructural (US) remodeling within the NVU of cerebral cortical gray matter and transitional subcortical white matter from db/db mice relative to non-diabetic wild-type age- and gender-matched mice on the same background [56,57]. Moreover, they observed cortical gray matter NVU, neuroglia, and myelin injury with US remodeling. The reconstruction of this unit seems to be crucial for recovery after stroke [58,59,60]. SGLT2 inhibition utilizing EMP prevented NVU’s cell and myelin US remodeling consisting of attenuation or loss of EC tight and adherent junctions of the BBB and various aberration including ECs and cortical matter [61]. There are no proves that EMP nor other SGLT2 can cross BBB, however SGLT2 inhibitors are lipid-soluble and should cross it [62]. Furthermore, there are reports that integrity and function of blood–brain barrier (BBB) are impaired during acute stroke phase [63]. Some authors hypothesized that empagliflozin can exert its neuroprotective effect by penetrating disrupted BBB [64]. In support, Hayden et al. [61] showed that empagliflozin could ameliorate ultrastructural remodeling of the neurovascular unit and neuroglia the brain of diabetic mouse, which emphasized the ability of empagliflozin to enter these regions where BBB has lost its integrity. Other researchers claim that it is more plausible to believe that SGLT inhibitors, in this case dapagliflozin, may attribute to increased GLP-1 concentrations, which can then cross the blood–brain barrier and lower corticosterone concentrations leading to neuroprotective effects [65].

## 4. Anti-Inflammatory Properties of SGLT2i May Slow Down Atherogenesis and Prevent Neuronal Loss Related to Oxidative Stress

Stroke can be prevented by eliminating risk factors, including carotid atherosclerosis [66]. It is a chronic inflammation of the blood vessels that causes plaque formation and subsequent narrowing of arteries [67]. Various cytokines are involved in the induction of inflammation-related atherosclerosis including: tumor necrosis factor alpha, IL-6 and monocyte chemotactic protein-1 (MCP-1), as well as media inducing expression and cell adhesion molecule 1 (VCAM-1) [68]. Systemic inflammation may disturb the integrity of blood–brain barrier leading to migration of proinflammatory agents to the CNS [69]. Consequent chronic low-grade inflammation has been proven to promote neuronal loss [70,71,72,73].

There are reports on possible relevance of SGLT2 inhibition to slowing down the development of atherosclerotic disease, which is one of the major causes of cerebral ischemic events. Han et al. [74] found that empagliflozin limits areas of atherosclerotic plaque in the aortic arch and valve compared to the control group I with glimepiride in ApoE-/-mice. The concentration of TNF-α, IL-6, and MCP-1 decreased after empagliflozin treatment, which was significantly correlated with the size of the plaque.

IL-6 and TNF-α are pro-inflammatory factors, the elevated concentrations of which are observed, inter alia, during stroke [75,76]. There are reports that TNF-α and IL-6 may increase the risk of stroke [77,78]. It has been supported by the work of Cui et al. [79] in a meta-analysis data acquired in Chinese population; however, Jefferis et al. [80] showed no such dependence on the British population. Similar results have been obtained by Pennig et al. [81] where atherosclerotic plaques in mice treated with empagliflozin were significantly smaller, simultaneously showing reduced lipid and a higher collagen content in their structure. Dimitriadis et al. [82] presented that empagliflozin diminished cholesterol levels, increased HDL (high density lipoproteins) cholesterol levels, and decreased the formation of atherosclerotic lesions and the expression of the inflammatory molecules VCAM-1 and MCP-1. Ganbaatar et al. [83] showed that treatment with empagliflozin significantly reduced the size of atherosclerotic lesions in the aortic arch and reduced lipid deposition and macrophage accumulation, and decreased the expression of MCP-1. Previously mentioned Hierro-Bujalance et al. [25] also observed reduced microglia burden in the parenchyma of db/db and APP/PS1xdb/db mice treated with EMP. However, the assessment has been proceeded in the limited range, by Iba1 immunostaining, not covering the whole complexity of the phenomenon.

Another SGLT2 inhibitor, canagliflozin has proven to be an inhibitor of endothelial cells (ECs) proliferation and DNA synthesis [84]. The process of controlling EC growth has been shown to play a pivotal role in atherogenesis inhibition [85]. The anti-proliferative action of canagliflozin was observed in ECs derived from mouse arterial circulation as well as from the human arterial and venous circulation. Based on lactate dehydrogenase activity measurements, the mechanism of its action may have rather cytostatic than cytotoxic character. The action seems to be concentration-dependent and appear at concentration reachable in the plasma of patients treated with the drug [86]. Moreover, the results imply that human cells may be more sensitive to the growth-inhibitory action of canagliflozin than mouse-derived ECs. Interestingly, this particular effect may be compound-specific effect, thus, pharmacologically relevant concentrations of other SGLT2 inhibitors, such as empagliflozin and dapagliflozin did not impact EC proliferation [87,88].

Amin et al. [64] presented results regarding SGLT2 treatment and oxidative stress limitation in rat CNS. Compared to gliclazide, empagliflozin administration lead to a decrease in infarct volume along with suppression of cerebral oxidative stress, inflammation and markers of apoptosis in rat brain tissue. The authors postulate that the neuroprotective effect of empagliflozin is primarily due to the reduction of oxidative stress. EMP decreased malondialdehyde (MDA), increased the activity of catalase and elevated glutathione (GSH) concentration in brain tissues. MDA is a marker for oxidative stress [89]. Catalase is an enzyme responsible for decomposition of hydrogen peroxide (source of reactive oxygen species (ROS)) into water and oxygen [90] and GSH is antioxidant that can prevent damaging cellular components caused by ROS [91]. Despite all of the promising results of experimental research presented in the paper, clinical efficacy of SGLT2i in cerebral ischemic events remains controversial. The meta-analysis by Zinman et al. [92] in patients with type 2 diabetes at high cardiovascular risk showed no significant difference in cerebrovascular risk including stroke after administration of empagliflozin compared to placebo. In the EMPA-REG OUTCOME trial, there was a slight, but not statistically significant, trend toward stroke risk elevation (HR, 1.18; 95% CI, 0.89 × 10^1.56^; P ¼ 0.26), possibly related to increased hematocrit [93]. The opposite trend has been obtained in the CANVAS trial of canagliflozin (HR, 0.87; 95% CI, 0.69 × 10^1.09^), although statistical significance, once more, has not been reached [94]. Summarized results of research described in the paper have been presented in Table 1.

## 5. Conclusions

The scope of available information in the literature is unfortunately limited. The results in experimental models are promising and mark another direction for the development of research in the field of therapeutic solutions for the treatment of AD and other conditions with primarily or secondarily neurodegenerative background. Nevertheless, there is a compelling need for further research in this area. Only the evaluation of clinical effectiveness will significantly answer the question whether drugs affecting SGLT2 transmission can effectively help in the treatment of CNS diseases. Collected data constitute one step further to full understanding of this multifactorial process of neuroprotection.

## Figures and Tables

**Table 1:** Summary of reviewed results.

Authors	Subject of Study	Type of SGLT2i	Dose of SGLT2i	Result
Abdel-Latif et al. [29]	Wistar rats	empagliflozin	1 and 10 mg/kg, p.o. twice after 1 and 24 h of reperfusion	↓ neurological defects, ↑ HIF-1α, ↑ VEGF-A, ↓ caspase-3, ↓ infract volume
Amin et al. [41]	Wistar rats	empagliflozin	1 and 10 mg/kg, p.o. twice after 1 and 24 h of reperfusion	↓ infract volume, ↓ MDA, ↑ catalase, ↑ GSH compared to gliclazide
Arafa et al. [22]	Wistar rats	canagliflozin	10 mg/kg p.o. once	↓ memory dysfunction
Behnammanesh et al. [63]	HUVECs, HAECs, MAECs	canagliflozin, empagliflozin, dapagliflozin	1–50 μM	↓ ECs proliferation
Ganbaatar et al. [62]	ApoE-/- mice	empagliflozin	20 mg/kg/day for 8 or 12 weeks	↓ size of atherosclerosis lesions, ↓ lipid deposition, ↓ macrophage accumulation, ↓ MCP-1
Han et al. [53]	ApoE-/- mice	empagliflozin	3 mg/kg/day for 8 weeks	↓ TNF-α, ↓ IL-6, ↓ MCP-1, ↓ atherosclerotic plaque
Hayden et al. [75]	db/db mouse	empagliflozin	10 mg/kg/day for 10 weeks	prevented NVU’s cell and myelin US remodeling
Hierro-Bujalance et al. [11]	APP/PS1xdb/db mice	empagliflozin	10 mg/kg/day for 22 weeks	↓ SP, ↓ Aβ, ↓ NOD, ↑ MWM, ↓ microglia burden
Lin et al. [17]	db/db mice	empagliflozin	diet containing 0.03% empagliflozin for 7 days or for 10 weeks	prevented cognitive function impairment, ↑ BDNF
Pennig et al. [60]	C57BL/6J mice	empagliflozin	35 mg/kg/day for 3 weeks	↓ size of atherosclerotic plaques, ↓ lipid and ↑ collagen content in atherosclerotic plaques structure

Note:↓—reduction, ↑—increase, GSH—glutathione, MDA—malondialdehyde, SP—senile plaques, Aβ—amyloid β, HIF-1α—hypoxia-inducible factor 1α, BDNF—brain-derived neurotrophic factor, VEGF-A—vascular endothelial growth factor-A, IL-6—interleukin 6, TNFα—tumor necrosis factor α, VCAM-1—vascular cell adhesion protein, MCP-1—monocyte chemotactic protein-1, ECs—endothelial cells, NVU—neurovascular unit, NOD—new object discrimination, MWM-Morris water maze.
